# Heterometallic Metal-Organic Framework Based on [Cu_4_I_4_] and [Hf_6_O_8_] Clusters for Adsorption of Iodine

**DOI:** 10.3389/fchem.2022.864131

**Published:** 2022-04-29

**Authors:** Huancheng Hu, Fangyun Chen, Zhanyun Zhang, Dongcheng Liu, Yuning Liang, Zilu Chen

**Affiliations:** State Key Laboratory for Chemistry and Molecular Engineering of Medicinal Resources, School of Chemistry and Pharmaceutical Sciences, Guangxi Normal University, Guilin, China

**Keywords:** heterometallic metal-organic framework, [Cu4I4] cluster, [Hf6O8] cluster, adsorption, iodine

## Abstract

Heterometallic metal-organic framework (MOF) as a kind of porous material is very important because of its excellent properties in catalysis, magnetic, sensor, and adsorption fields, but the reasonable design and syntheses of these are still challenging. Herein, we prepared one heterometallic MOF with the formula [Hf_6_(*μ*_3_-OH)_8_(OH)_8_][(Cu_4_I_4_) (ina)_4_]_2_·22DMF (**NS-1**, ina = isonicotinate). Single-crystal X-ray diffraction analysis revealed that **NS-1** is a three-dimensional network with **flu** topology, constructed from 8-connected [Hf_6_(*μ*_3_-OH)_8_(OH)_8_]^8+^ and 4-connected [Cu_4_I_4_] clusters as second building units (SBUs). To our best knowledge, **NS-1** is a rare example with two different metal clusters as SBUs in heterometallic Hf-based MOFs. Interestingly, **NS-1** exhibits a reversible adsorption performance for iodine in the cyclohexane solution, the adsorption kinetics fits well with the pseudo-second-order equation, and the Freundlich model relating to multilayer adsorption better describes the process of iodine absorption.

## Introduction

Iodine, which is a key raw material to synthesize thyroid hormones, plays an important role in the development of human nervous system. Once the content of iodine is lower than the normal value in the human body for a long period, people will suffer from iodine deficiency disorders. Meanwhile, the ingestion of excess iodine also can damage the health of humans (He et al., 2021; Xian et al., 2022). Nuclear technology has witnessed the development of society, but the disposal of nuclear waste becomes a worldwide issue owing to the occurrence of some accidents related to nuclear release. Taking radioactive iodine (^129^I and ^131^I) as an example, the half-time (*t*_1/2_) of ^129^I is 1.57 × 10^7^ years and *t*_1/2_ = 8.02 days for ^131^I; when these species enter into the environment, they will threaten the health of plants, animals, and humans (Yin et al., 2012; Luo et al., 2022). Thus, the design and preparation of materials which can effectively adsorb iodine becomes a significant research field for scientists. In the past decades, various adsorbent materials (e.g., porous carbon, silver-containing mordenite, and molybdenum sulfide porous chalcogel) exhibited the adsorption capacity of iodine (Chapman et al., 2010; Subrahmanyam et al., 2015; Sun et al., 2019); however, these materials often possessed relatively low porosity compared with that of MOFs, leading to lower adsorption capacity than MOFs.

In the past two decades, hafnium-based metal-organic frameworks (Hf-MOFs) have been given much attention because of their fascinating structures, high surface area, and excellent chemical and thermal stabilities, which provide the theoretical basis for their promising applications in catalysis, sensing, adsorption and separation, drug delivery, etc (Hu et al., 2016; Cao et al., 2017; Abánades Lázaro and Forgan, 2019; Angeli et al., 2020). For example, Hf_6_(*μ*_3_-O)_4_ (*μ*_3_-OH)_4_(L)_6_ (H_2_L = 9,10-anthacenyl bis(benzoic acid)) could serve as an efficient X-ray scintillator (Wang et al., 2014), the partially dehydrated **Hf-NU-1008** exhibited high catalytic activity of CO_2_ cycloaddition of styrene oxide (Lyu et al., 2019), [Hf_6_O_4_(OH)_4_ (ADC)_6_] (ADC = acetylenedicarboxylate) and [Hf_6_O_4_(OH)_4_(Fum-Cl)_6_] (Fum-Cl = chlorofumarate) show good water vapor absorption, and the latter also possesses the properties of adsorbing CO_2_ and molecular iodine vapor (Matemb Ma Ntep et al., 2019). But, heterometallic Hf-MOFs have been rarely reported. In addition, [Cu_*x*_I_*y*_] cluster-based MOFs such as {[(CuI)_2_ (TPP)]·solvent}_*n*_ and {[(Cu_2_I_2_) (TPP)]·solvent}_*n*_ (TPP = tetra-4-(4-pyridyl)phenylmethane) (Kitagawa et al., 2013) and [Tb(Cu_4_I_4_) (INA)_3_ (DMF)] (INA = isonicotinate) (Hu et al., 2017) also exhibit good adsorption behavior of iodine. According to the Hard–Soft-Acid–Base theory, Hf(IV) usually forms coordination bonds with oxygen atoms to build the [Hf_*x*_O_*y*_] cluster, especially for the [Hf_6_O_8_] core, while Cu(I) can coordinate with N and O atoms. Therefore, to gain stable heterometallic Hf-MOFs with the high adsorption value of iodine, we choose isonicotinic acid as a ligand to construct and prepare the heterometallic metal-organic framework featuring [Hf_6_O_8_] and [Cu_4_I_4_] clusters.

In this context, we herein choose isonicotinic acid as the organic linker and HfCl_4,_ and CuI as the metal source, preparing one rare heterometallic cluster-based Cu-Hf metal-organic framework. The structure, characterization, iodine adsorption, and release experiments of **NS-1** have been investigated in detail.

## Experimental Section

### Materials

All chemicals in our study were purchased from commercial companies and used without further purification.

### Synthesis of NS-1

A mixture of isonicotinic acid (49.2 mg, 0.4 mmol), HfCl_4_ (32.0 mg, 0.1 mmol), CuI (19.0 mg, 0.1 mmol), DMF (4.00 ml), and HCOOH (2.05 ml) were added into teflon-lined stainless steel vessel, placed in an oven at 120°C for 2 days, and cooled down to room temperature at the rate of 2.5°C/h. Yellowish polyhedron-shaped crystals were collected by filtration and washed three times with fresh DMF.

### Characterization

Single-crystal X-ray diffraction data were recorded on a Supernova diffractometer (Rigaku, Japan) equipped with graphite monochromated and Mo−Kα radiation (*λ* = 0.71073 Å). Powder X-ray diffraction (PXRD) patterns were collected using a D/Max-3c X-ray diffractometer (Rigaku, Japan). Four transform infrared spectroscopy (FT-IR) spectra were performed on a Spectrum Two spectrometer (PerkinElmer, United States). High-resolution mass spectrometry was performed on an Exactive mass spectrometer (Thermo Fisher Scientific, Germany). Thermogravimetric analysis (TGA) was performed on a Labsys evo TG-DTA/DSC analyzer (Setaram instrument, France) under an N_2_ atmosphere from 25 to 1000°C with the heating rate of 10°C min^−1^. UV−Vis absorption spectra were recorded on a CARY ECLIPSE JASCO-720 spectrophotometer (Agilent, United States). Raman spectra were measured on a Renishaw Invia Raman spectrometer (Invia, United Kingdom).

### X-Ray Crystallography

The structure of **NS-1** (CCDC 2149625) was solved *via* the direct method and refined using full-matrix least-squares on *F* ^2^ by the SHELXTL and OLEX2 program packages (Dolomanov et al., 2009; Bourhis et al., 2015; Sheldrick, 2015). All nonhydrogen atoms were refined in anisotropic approximation, and all H atoms were treated with the riding model. Heavy disordered solvent molecules exist in the unit cells of **NS-1**; thus, SQUEEZE (PLATON) has been treated as a diffuse contribution to the overall scattering without specific atom positions. The electron counts in the voids calculated by PLATON SQUEEZE could roughly speculate the number of guest molecules in **NS-1** (Sudik et al., 2005; Seo et al., 2011).

### Iodine Adsorption of NS-1

At room temperature, 10.0 mg of fresh **NS-1** was immersed in 5 ml cyclohexane solution of iodine with different initial concentrations (300–700 mg/L) under magnetic stirring. The residual concentration of iodine in cyclohexane was monitored by a UV–Vis spectrophotometer.

### Iodine Desorption of Iodine-Loaded NS-1 (NS-1′)

1.0 mg of **NS-1** was soaked in an iodine-dissolved cyclohexane solution (300 mg/L, 5 ml) for 5 h to obtain **NS-1′** under room temperature and then the sample was immersed into 10 ml ethanol, and the release rate of iodine was detected by UV–Vis spectrophotometer.

## Results and Discussion

### Structural Description of NS-1

**FIGURE 1:**
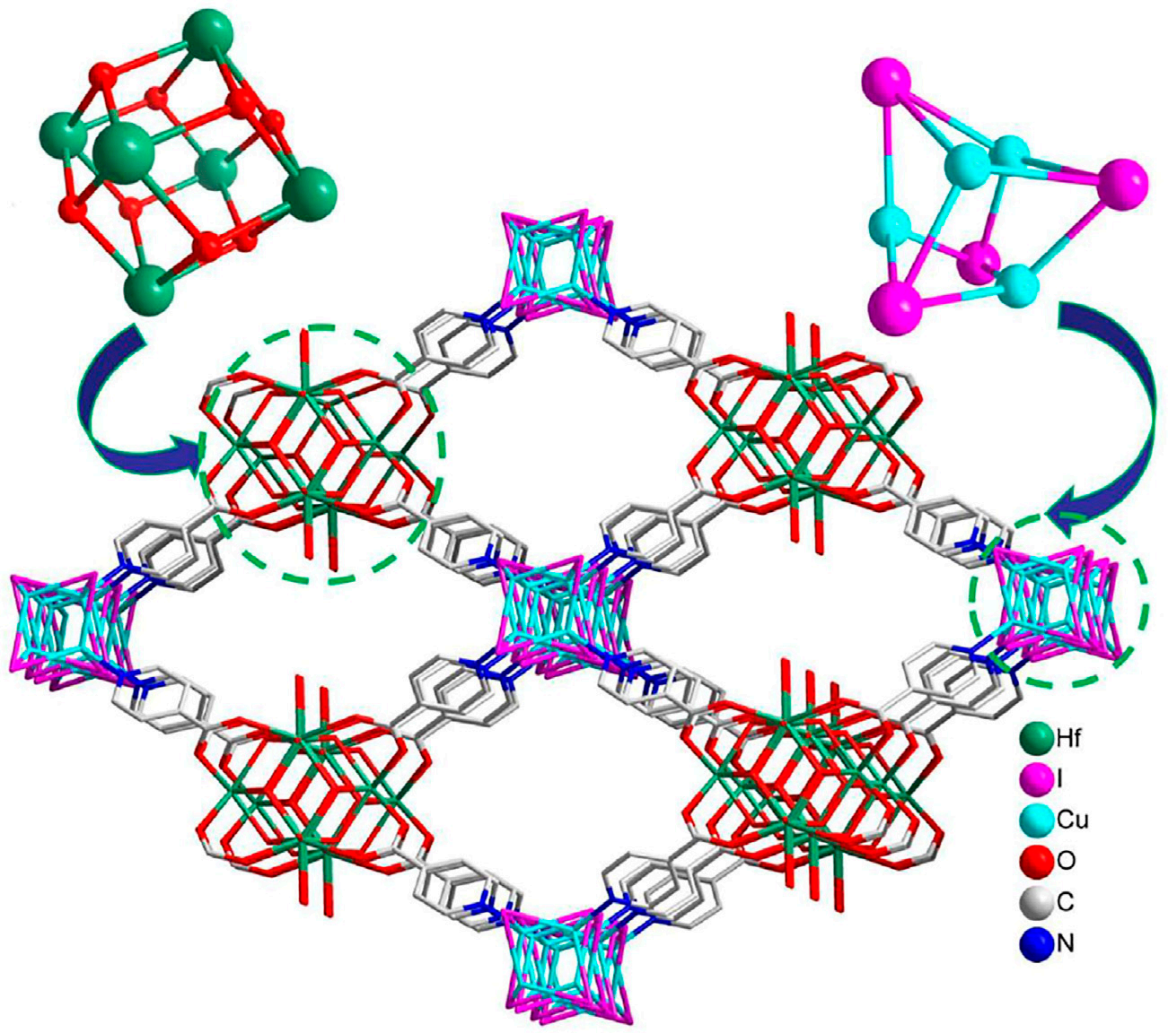
Three-dimensional network of **NS-1**; all hydrogen atoms were omitted for clarity.

Single-crystal X-ray diffraction analysis revealed that **NS-1** crystallizes in the tetragonal space group *I*4/mmm, containing two different second building units (SBUs) with 4-connected cube-like [Cu_4_I_4_] and 8-connected dodecahedral [Hf_6_(*μ*_3_-OH)_8_(OH)_8_] clusters, which is the isostructure with [Zr_6_ (*μ*_3_-OH)_8_(OH)_8_][Cu_4_I_4_(Ina)_4_]_2_·*x*guest (Tan et al., 2016). The Hf1 is surrounded by eight O atoms, which are derived from four *μ*_3_-OH and four isonicotinate anions; the Hf2 is also coordinated with eight O atoms, stemming from four *μ*_3_-OH, two OH^−^ and two isonicotinate anions. Two Hf1 atoms are located in the axial plane, and four Hf2 atoms are located in the equatorial plane to build an octahedral [Hf_6_] core. The triangular planes of the [Hf_6_] octahedron are alternatively capped by eight *µ*_3_-OH groups to construct a dodecahedral [Hf_6_(*μ*_3_-OH)_8_] fragment. Each Cu center coordinates with three I^−^ ions and one N atom from an isonicotinate anion, leading to tetrahedral geometry. Four Cu atoms and four I^−^ ions construct a cube-like [Cu_4_I_4_] unit. Six [Hf_6_(μ_3_-OH)_8_(OH)_8_]^8+^ clusters, eight [Cu_4_I_4_] clusters, and 24 ina^−^ linkers can form a cage in **NS-1** (Supplementary Figure S1), and these cages are further packed together to build a 3D network (Supplementary Figure S2A). Each [Hf_6_(*μ*_3_-OH)_8_(OH)_8_]^8+^ cluster links to eight isonicotinate anions, each [Cu_4_I_4_] unit connects to four isonicotinate anions, and [Hf_6_(*μ*_3_-OH)_8_(OH)_8_]^8+^ and [Cu_4_I_4_] units are bridged together with one isonicotinate anion, resulting in a 4,8-connected network with **flu** topology (Supplementary Figure S2B). It is noteworthy that this type of topology has been reported in some pure Zr/Hf-based MOFs, which were constructed from the 8-connected [M_6_ (μ_3_-OH)_8_(OH)_8_]^8+^ (M = Zr and Hf) fragment and 4-connected organic linker, such as **MOF-841** (Furukawa et al., 2014), **PCN-902-X** (X = O and CH_2_) (Wang et al., 2019), and **MFM-133** (Yan et al., 2018), while it is rare for heterometallic Zr/Hf-based MOFs. Noteworthily, compared with the reported heterometallic Hf-based MOFs, **NS-1** is an unusual example with different metal clusters as SBUs. In addition, the 3D network is packed with a rhombus channel of approximately 13.10 × 19.27 Å^2^ (Figure 1). Moreover, combining with some previous MOFs with different metal cores as SBUs, such as **tp-PMBB-1-asc-1** based on trigonal-prismatic clusters [Cr_3_O(isonic)_6_(H_2_O)_3_]^+^ (isonic = pyridine-4-carboxylate) and Zn^2+^ cations (Schoedel et al., 2013), **rht**-MOFs are built from copper paddlewheel [Cu_2_(O_2_C)_4_] and triangular inorganic [Cu_3_O(N_4−*x*_ (CH)_*x*_C)_3_] (*x* = 0, 1, or 2) units (Gao et al., 2015) and **FDM-4−FDM-8** feature with dinuclear [Zn_2_]/tetranuclear [Zn^II^_4_O] and triangular Cu^I^_3_(NN)_3_/Cu^II^_3_(*µ*_3_-OH) (NN)_3_ cores (Tu et al., 2017; Tu et al., 2019); thus, selecting the organic molecules with both N and O atoms (eg. isonicotinate acid, 4-pyrazolecarboxylic acid, 5-tetrazolylisophthalic acid, 5-trizolylisophthalic acid) or mixing different kinds of multidentate organic linkers may be viewed as an effective synthetic strategy in the construction of heterometallic MOFs.

### Chemical Stability of NS-1

The powder X-ray diffraction (PXRD) pattern of the as-synthesized **NS-1** was recorded, and it can match well with the simulated ones from single-crystal X-ray diffraction data, confirming the good purity of **NS-1**. Moreover, in order to explore the stability of **NS-1** in cyclohexane solution, 50 mg of fresh **NS-1** was soaked in cyclohexane solution for 12 h under room temperature, and then the PXRD pattern of the sample was measured, which is consistent with the as-synthesized ones (Supplementary Figure S3). In addition, high-resolution mass spectrometry (HRMS) test was carried out on the remaining solution after **NS-1** was immersed in cyclohexane, and no obvious signal was detected, which is indicative of absence of **NS-1** and the fragments related to **NS-1** in the remaining solution. Both the PXRD pattern and HRMS test reveal the good stability of **NS-1** after being soaked in cyclohexane solution.

### Thermogravimetric Analysis of NS-1

The weight loss of **NS-1** from room temperature to 276°C is 29.9%, which corresponds to the loss of DMF molecules in channels, being identical to the calculated 29.6% (Supplementary Figure S4); thus, **NS-1** exhibited high thermal stability.

### Iodine Adsorption of NS-1

**FIGURE 2:**
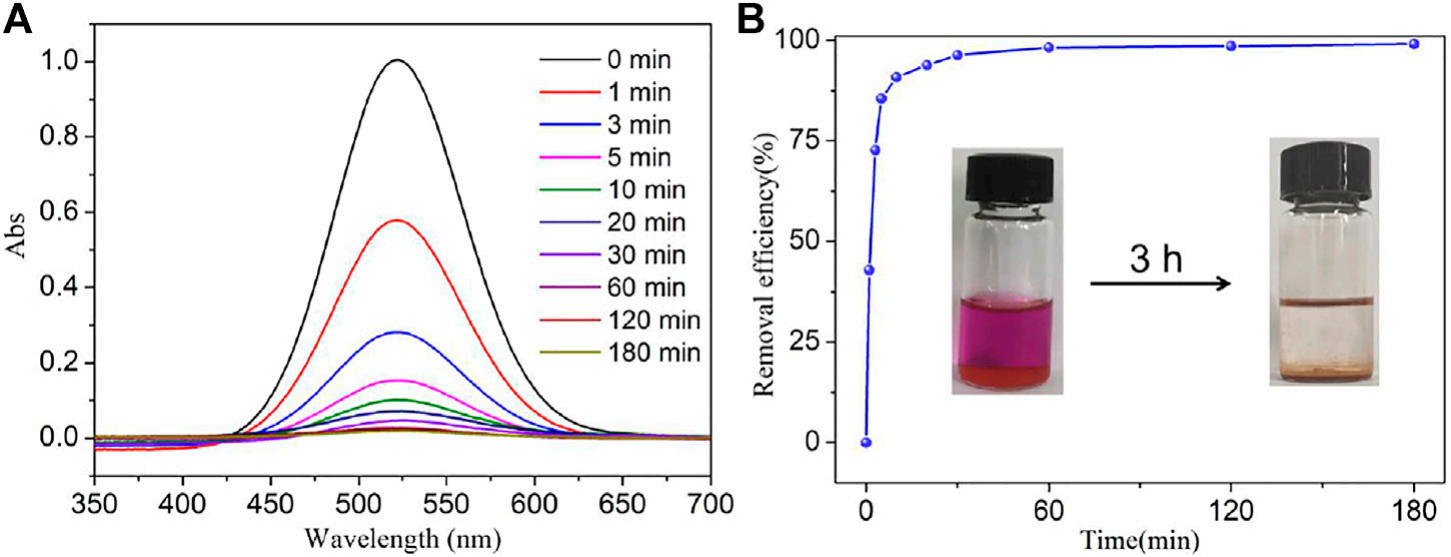
**(A)** Temporal evolution of UV–Vis absorption spectra for the adsorption of iodine by **NS-1** in cyclohexane and **(B)** the rate of iodine adsorption in **NS-1** in cyclohexane (*C*_0_ = 300 mg/L).

[Cu_*x*_I_*y*_] cluster-based MOFs usually exhibit good iodine adsorption capacity because of their strong interaction between the [Cu_*x*_I_*y*_] core and iodine (Kitagawa et al., 2013). **NS-1** possesses a large channel of 13.10 × 19.27 Å^2^ and provides the possibility of the iodine molecules with a diameter of 3.35 Å into the voids. Inspired by these features of **NS-1**, the iodine adsorption experiment of **NS-1** was carried out. As shown in the inset of Figure 2B and Supplementary Figures S6B–S9B, the color of the iodine solution gradually faded, while the color of **NS-1** changed from light yellow to brown. In addition, the absorbance of iodine solution decreased with the iodine absorbed by **NS-1** (Figure 2A). These behaviors indicate that iodine molecules were adsorbed by **NS-1.**

In order to determine the capture value of iodine *Q*_*t*_ (mg/g) and the iodine removal efficiency (*R*%) at any arbitrary time, the standard curve of iodine dissolved in cyclohexane was prepared by the following method: a 1,000 mg/L iodine solution was obtained by dissolving 50 mg iodine in 50 ml cyclohexane solution, and it was diluted to different concentrations using cyclohexane; the absorbance of these solutions was recorded by a UV–Vis spectrophotometer. As depicted in Supplementary Figure S5, the absorbance of 522 nm obviously increases with the increasing concentrations of iodine solution; as a result, the relationship between absorbance and concentration of iodine solution can be represented by the equation of *y* = 0.01032 + 0.00344*x*, where *y* is the absorbance of iodine solution and *x* is the concentration of iodine solution. Thus, we can calculate the concentration of iodine solution at an arbitrary time *C*_*t*_ (mg/L) after being absorbed by **NS-1** *via* measuring the absorbance of the iodine solution. The values of *Q*_t_ and *R*% were calculated by the equations $Q_{t}=\frac{\left(C_{0}-C_{t}\right)\times V}{m}$ and $R\%=\frac{C_{0}-C_{t}}{C_{0}}\times 100\%$ , where *C*_0_ (mg/L) is the initial concentration of iodine solution, *V* (ml) represents the volume of iodine solution, and *m* (mg) is the mass of **NS-1** (Qian et al., 2016; Chen H. et al., 2020). As shown in Figure 2B and Supplementary Figures S6B–S9B, the removal rate of iodine in cyclohexane solution by **NS-1** increased quickly in the primary 20 min, and it could obtain 90%, especially for the initial concentration of 300 mg/L iodine solution, and it only takes 10 min to gain a removal efficiency of 90%, and then the removal rate gradually decreased and finally reached the equilibrium adsorption around 1 hour. The equilibrium adsorption amount and removal efficiency of iodine in the cyclohexane solution are summarized in Supplementary Table S2. When the initial concentration of the iodine solution is 700 mg/L, the equilibrium uptake is 329 mg/g, corresponding to the removal efficiency of 92.0%. The removal performance of iodine is better with MIL-101-NH_2_ (311 mg/g, 90%, 30 h) (Falaise et al., 2013), ZIF-8@PU (330 mg/g, 90%, 96 h) (Zhao et al., 2019), and UiO-66 (401 mg/g, 43.5%, 24 h) (Wang et al., 2017) and lower than that of UiO-66-PYDC (1,250 mg/g, 98.3%, 24 h) (Wang et al., 2017).

To investigate the iodine adsorption kinetics of **NS-1**, two common kinetic models (the pseudo-first- and pseudo-second-order kinetic models) were considered, and their corresponding equations are as follows:

$$Q_{t}=Q_{e}-Q_{e}\times e^{-k_{1}t}\;\;\;\left(Pseudo-first\;order\;kinetic\;\mathrm{mod}el\right),$$

$$\frac{t}{Q_{t}}=\frac{1}{k_{2}Q_{e}^{2}}+\frac{1}{Q_{e}}t\;\;\;\left(Pseudo-\sec ond\;order\;kinetic\;\mathrm{mod}el\right),$$

where *k*_1_ (min^−1^) and *k*_2_ (g mg^−1^ min^−1^) are, respectively, the pseudo-first- and pseudo-second-order rate constant for the adsorption process and *Q*_*e*_ (mg/g) is the equilibrium adsorption amount of iodine by **NS-1**.

**FIGURE 3:**
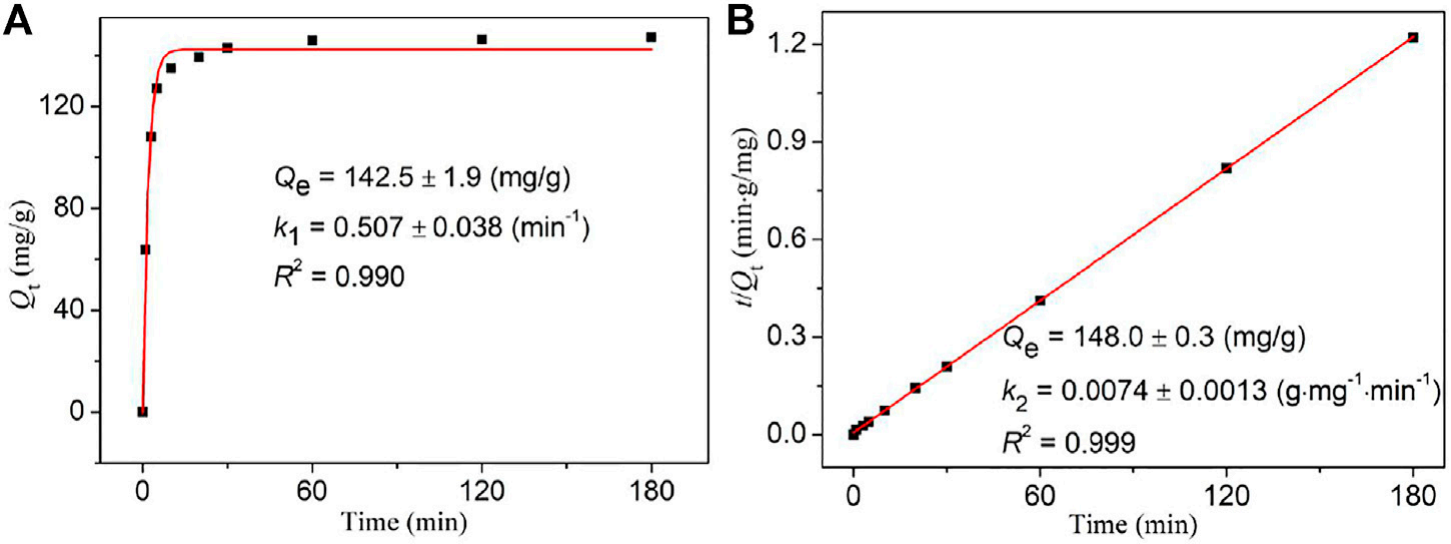
Pseudo-first **(A)** and pseudo-second order **(B)** kinetic models for the iodine adsorption kinetics of **NS-1** with the initial concentration of 300 mg/L cyclohexane solution.

As depicted in Figure 3, Supplementary Figures S10, S11, compared with the related fitting parameters obtained *via* the pseudo-first and pseudo-second order kinetic equations, the pseudo-second order kinetic equation better describes the iodine adsorption in cyclohexane by **NS-1**, revealing that multilayer chemisorption involving strong interactions between **NS-1** and iodine molecules exists in the absorption progress (Kameda et al., 2020; Yadollahi et al., 2020).

In order to further explore the iodine absorption isotherm of **NS-1**, we choose two common isotherm models (Langmuir and Freundlich isotherm model) to simulate with the experimental iodine adsorption isotherm. The related parameters and *R* ^2^ obtained *via* these two models are listed in Supplementary Table S3. These isotherm models are expressed by the following equations:

$$Q_{e}=\frac{Q_{m}K_{L}C_{e}}{1+K_{L}C_{e}}\;\;\;\left(Langmuir\;isotherm\;\mathrm{mod}el\right),$$

$$Q_{e}=K_{F}C_{e}^{1/n}\;\;\;\left(Freundlich\;isotherm\;\mathrm{mod}el\right),$$

where *C*_*e*_ (mg/L) is the equilibrium concentration of the iodine solution; *Q*_*m*_ (mg/g) is the theoretical maximum adsorption value; *K*_L_ (L/mg) and *K*_F_ (mg/g) represent the adsorption constants of Langmuir and Freundlich isotherm models, respectively; and *n* is the Freundlich linearity index.

**FIGURE 4:**
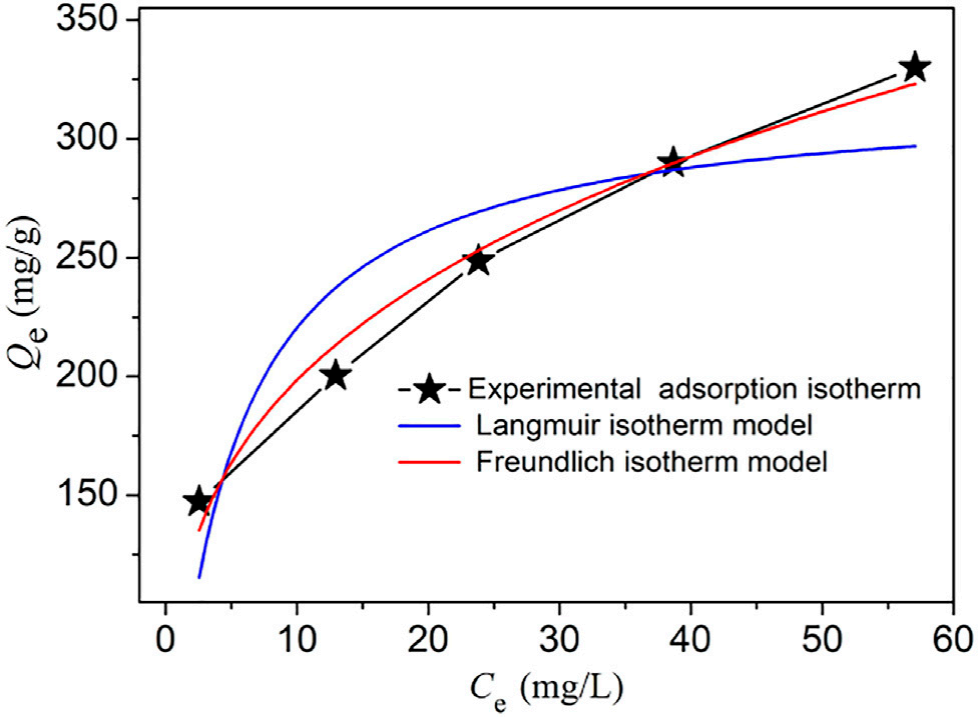
Experimental iodine adsorption isotherms (black line) and the corresponding fitting lines of Langmuir (blue line) and Freundlich (red line) isotherm models.

As shown in Figure 4 and Supplementary Table S3, the Freundlich isotherm model fits the experimental adsorption isotherm better than the Langmuir isotherm models, namely, the Freundlich isotherm model which is more reasonable to describe the iodine adsorption behavior of **NS-1**. So, the iodine adsorption process of **NS-1** may be attributed to the multilayer adsorption on the heterogenous surface of **NS-1**, which is coincident with the mechanism of iodine adsorption kinetics. This iodine adsorption process of **NS-1** is similar to some previously reported MOFs such as **Th-SINAP-8**, **MIL-101-NH**_**2,**_ and **CAU-1 (** Falaise et al., 2013; Li et al., 2020). Moreover, the 1/*n* value of 0.28 is located in the range of 0.1–0.5, which is indicative of the good iodine capture performance of **NS-1**.

To explore the possible species of iodine in **NS-1′**, Raman spectra of original **NS-1** and iodine-loaded **NS-1′** were recorded. As shown in Supplementary Figure S12, there is an obvious peak of 168 cm^−1^ for the original **NS-1**, which may be attributed to the Cu–I vibrations (He et al., 2014; Zhao et al., 2016). The peak occurring at 171 cm^−1^ for iodine-loaded **NS-1′** is greatly intensified and shifted compared to that of **NS-1**, and this peak is close to the vibrational frequency of iodine solid and H...I vibrations. Thus, it is probably ascribed to the iodine molecule located in **NS-1** and/or the formation of hydrogen bonds between the -OH from the network of **NS-1** and iodine molecules (Zhang et al., 2017; Chen P. et al., 2020).

**FIGURE 5:**
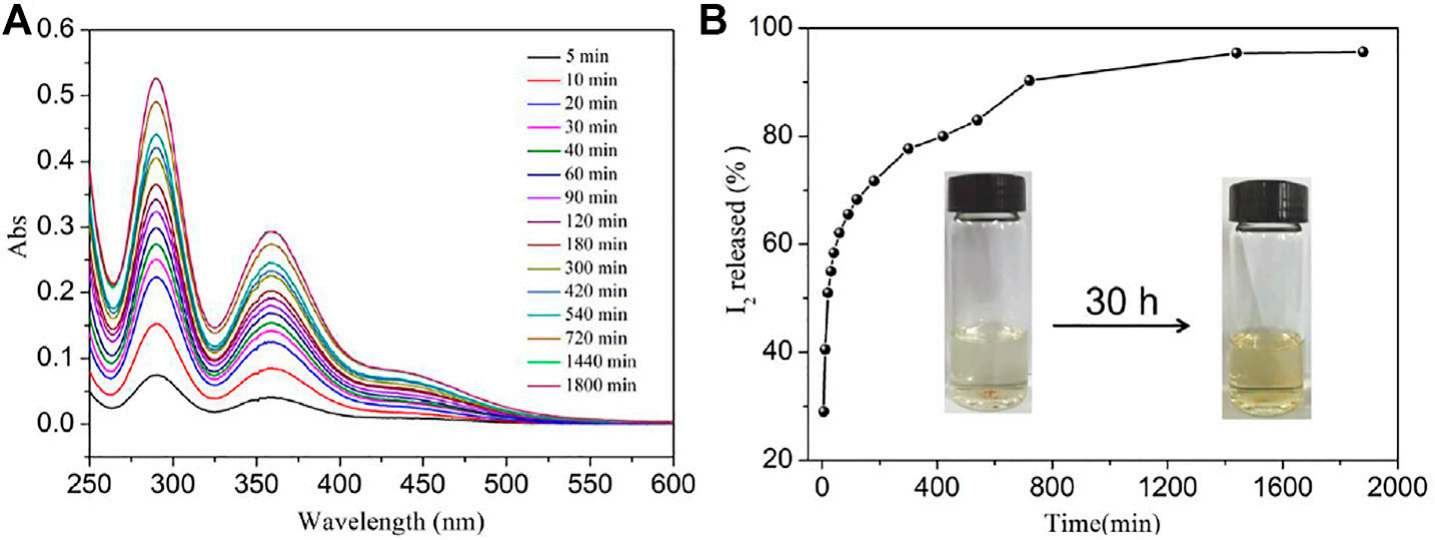
**(A)** Temporal evolution of UV–Vis absorption spectra for the release of iodine from **NS-1′** in ethanol and **(B)** the rate of iodine release from **NS-1′** in ethanol.

The reversibility of a potential iodine absorbent is very necessary; thus, the experiment of iodine releasing from iodine-loaded **NS-1** was investigated as the similar method of iodine adsorption by **NS-1** and the standard curve of iodine dissolved in ethanol as well was obtained (Figure 5 and Supplementary Figure S13). The absorbance of 290 and 360 nm increases with the soaking time, the release of iodine was very fast during the first 3 h and then the delivery of iodine became slow, and finally the release progress approached to the equilibrium state with a recovery rate of 95.4% after 24 h. This behavior implies that strong interactions exist between the network of **NS-1** and iodine**.** Meanwhile, the PXRD pattern of the recovered **NS-1** in ethanol was recorded, which can also agrees well with the as-synthesized ones, confirming the stability and renewability of **NS-1** (Supplementary Figure S3).

## Conclusion

A three-dimensional heterometallic metal-organic framework with [Hf_6_(*μ*_3_-OH)_8_(OH)_8_]^8+^ and [Cu_4_I_4_] clusters have been synthesized and characterized in detail, which is a rare example with two different metal clusters as second building units in heterometallic Hf-based MOFs. Interestingly, our experiments show that this framework exhibits excellent capability of iodine adsorption. The kinetics of this adsorption process are further studied. This work will broaden the vision of constructing and preparing heterometallic hafnium-based MOFs with more than one type of cluster and designing materials with high iodine adsorption capacity.

## Data Availability

The datasets presented in this study can be found in online repositories. The name of the repository, accession number, and DOI can be found below: Cambridge Structural Database; CCDC 2149625; DOI: 10.5517/ccdc.csd.cc2b4vr3.
