# Supplementary material for: Heterometallic Metal-Organic Framework Based on [Cu4I4] and [Hf6O8] Clusters for Adsorption of Iodine
Source: Front Chem. 2022 Apr 29;10:864131. doi: 10.3389/fchem.2022.864131 (PMC9098963; doi:10.3389/fchem.2022.864131)
Supplement: Supplementary file 1 [file DataSheet1.zip › Data Sheet 1/Supplementary_Material.docx]

Supplementary Material


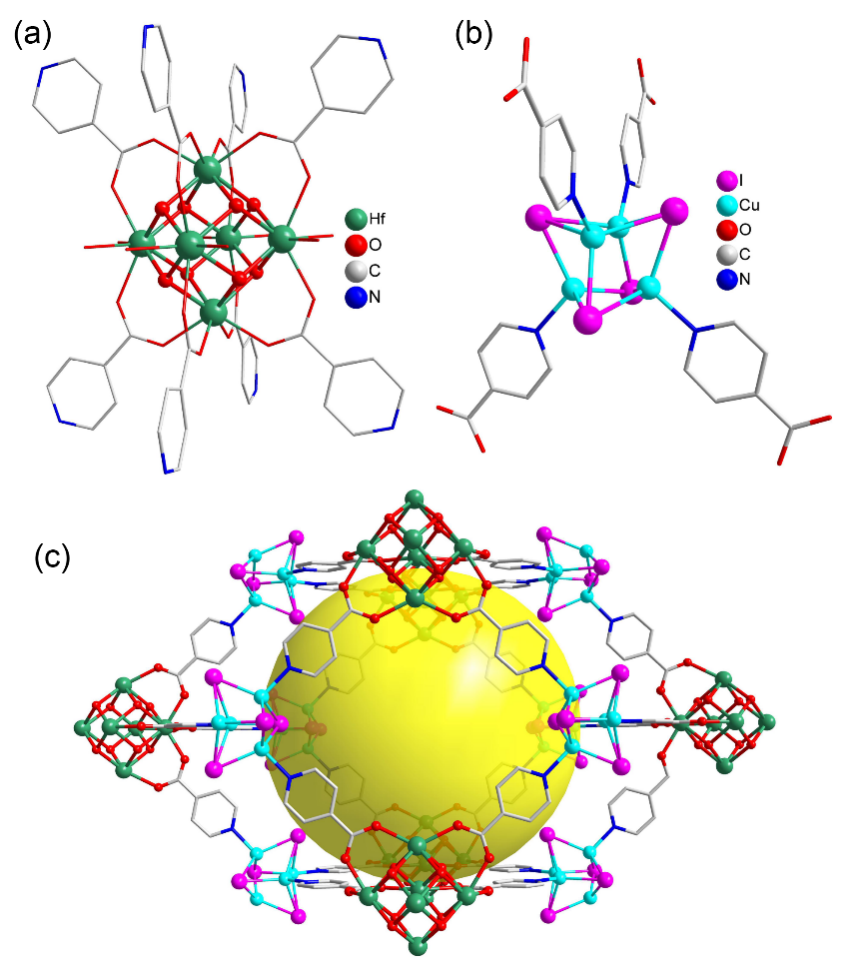


**Supplementary Figure S1.** (A) The 8-connected [Hf_6_(*μ*_3_-OH)_8_(OH)_8_(ina)_8_] cluster, (B) the 4-connected [Cu_4_I_4_(ina)_4_]^4-^ cluster and (C) the cage built by six [Hf_6_(*μ*_3_-OH)_8_(OH)_8_]^8+^ clusters, eight [Cu_4_I_4_] clusters and twenty-four ina^-^ linkers in **NS-1**.


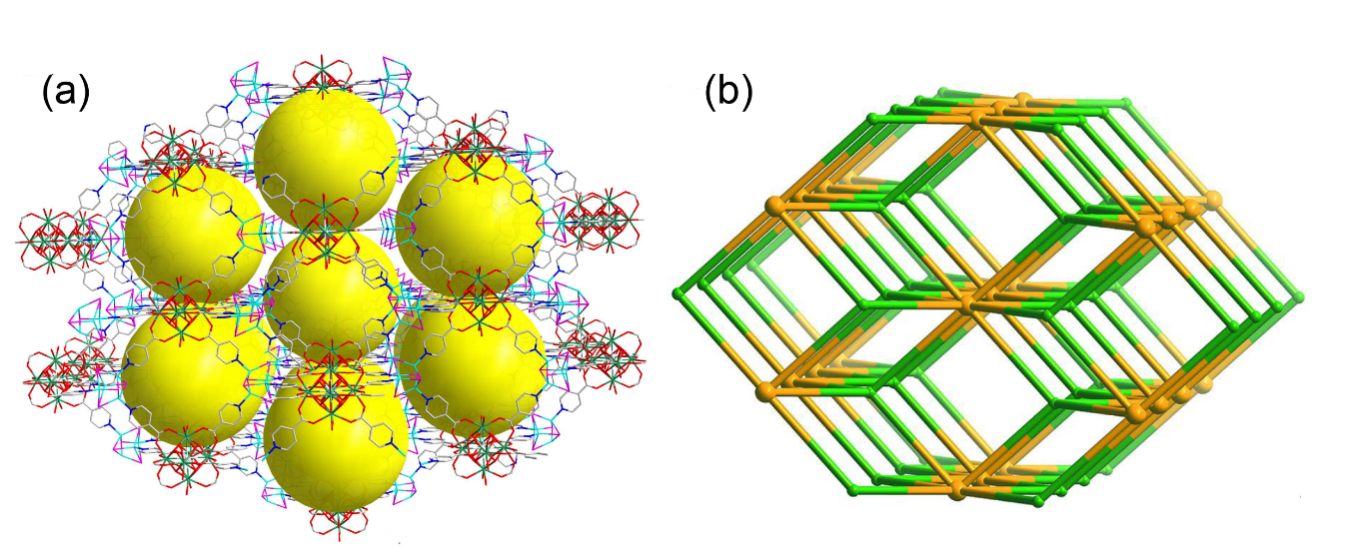


**Supplementary Figure S2.** (A) The 3D network and (B) The **flu** topology of **NS-1**.


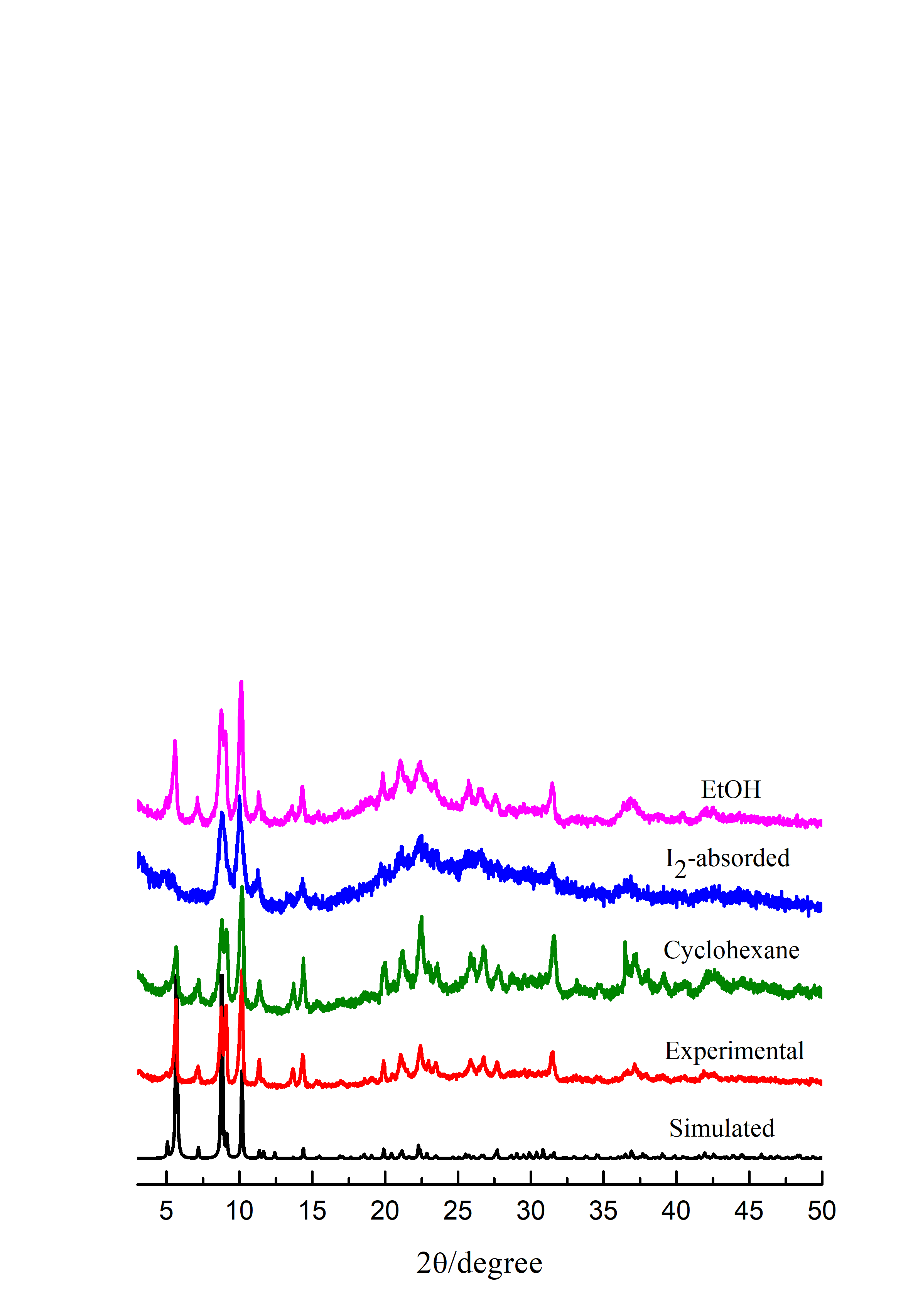


**Supplementary Figure S3.** Powder X-ray diffraction patterns of simulated and as-synthesized **NS-1**, **NS-1** after treated in cyclohexane, iodine-dissolved cyclohexane solution with 300 mg/L and **NS-1'** after treated in EtOH solution.


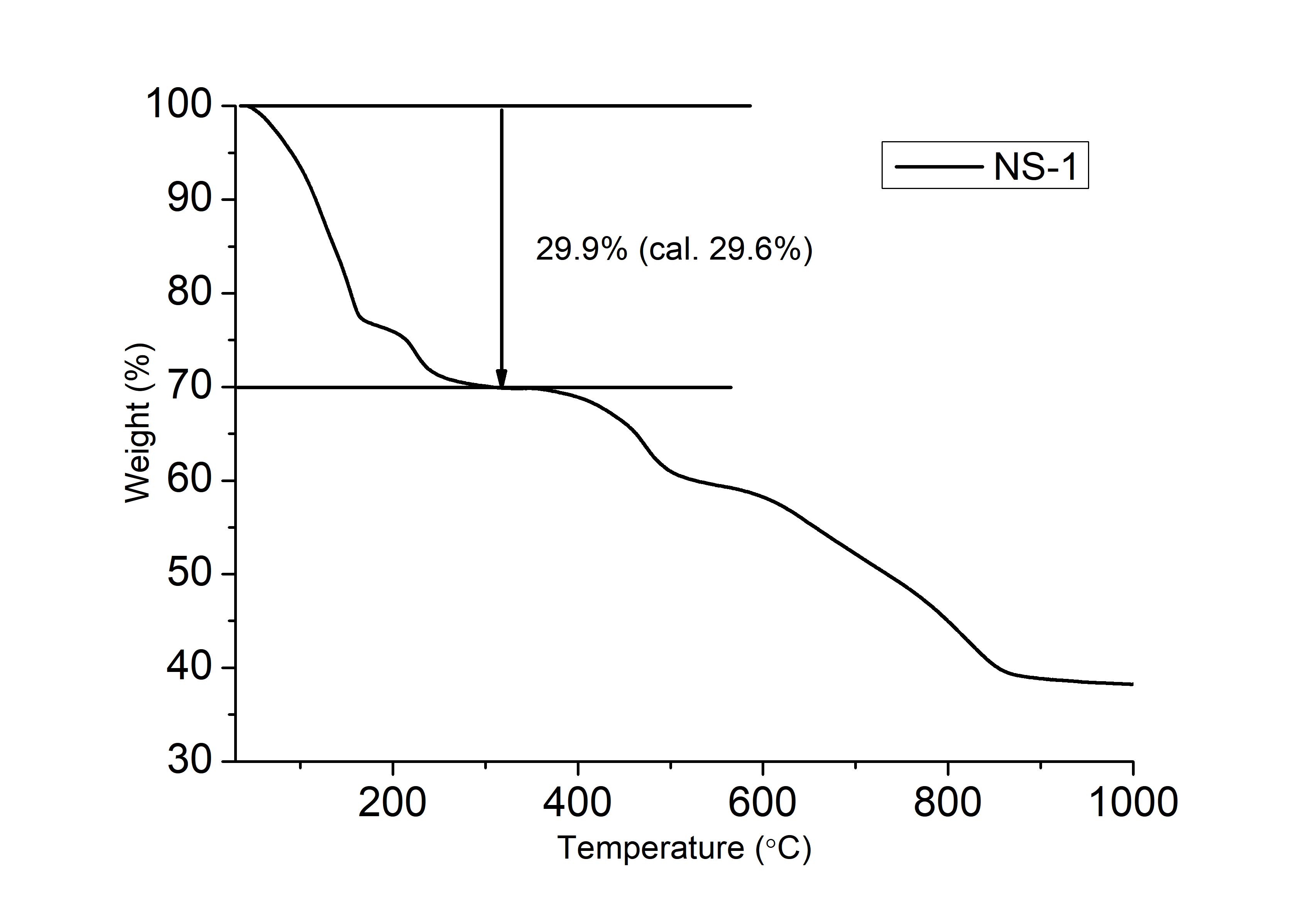


**Supplementary Figure S4.** Thermogravimetric analysis of **NS-1**.


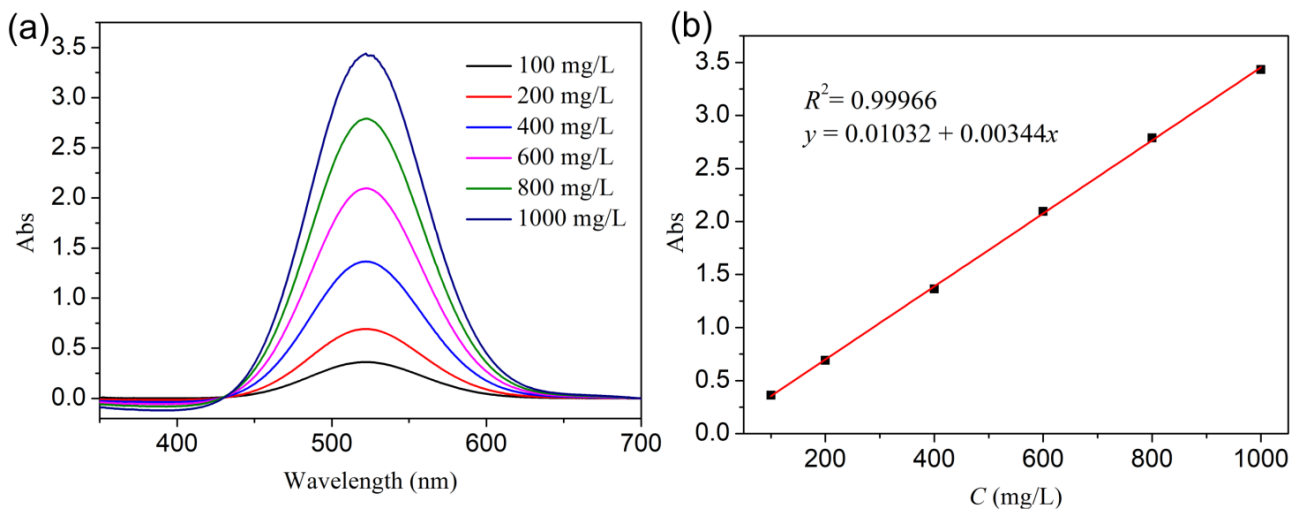


**Supplementary Figure S5.** The standard curves of iodine-dissolved in cyclohexane solution.


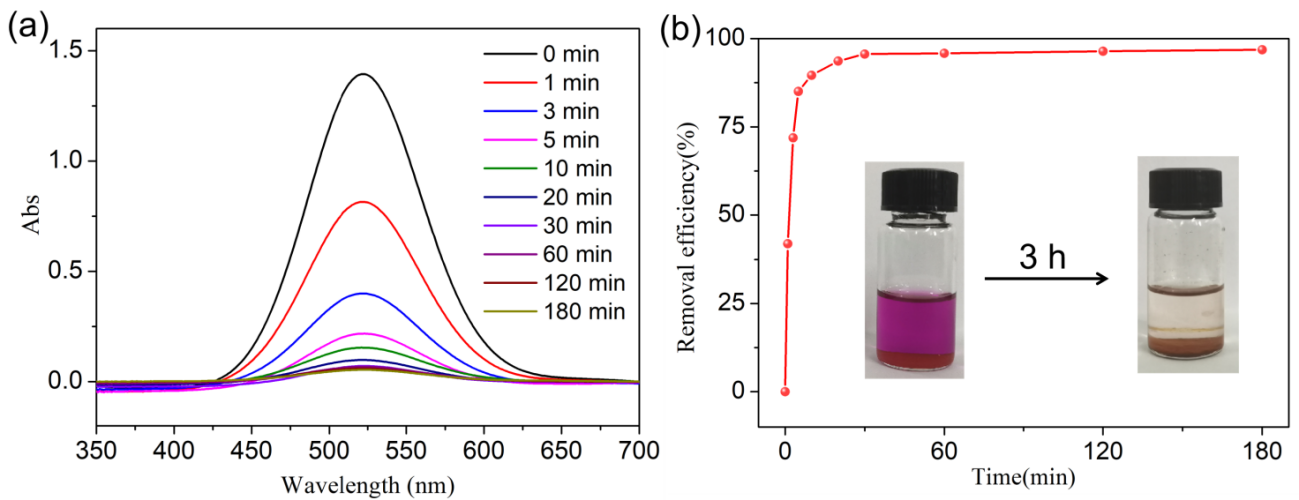


**Supplementary Figure S6.** (A) Temporal evolution of UV-vis absorption spectra for the adsorption of iodine by **NS-1** in cyclohexane and (B) the rate of iodine adsorption by **NS-1** in cyclohexane (*C*_0_ = 400 mg/L).


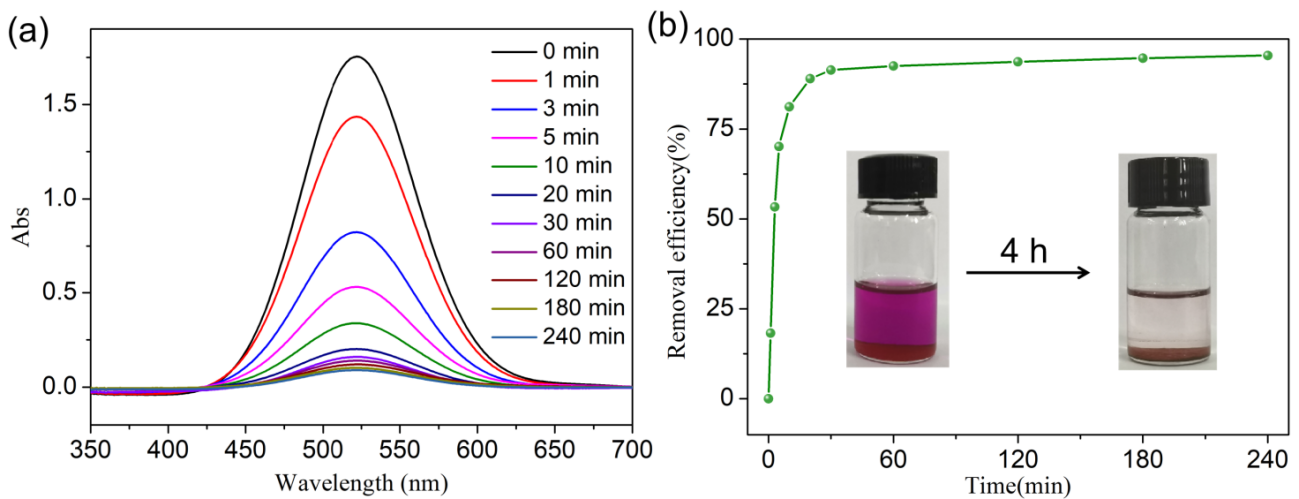


**Supplementary Figure S7.** (A) Temporal evolution of UV-vis absorption spectra for the adsorption of iodine by **NS-1** in cyclohexane and (B) the rate of iodine adsorption by **NS-1** in cyclohexane (*C*_0_ = 500 mg/L).


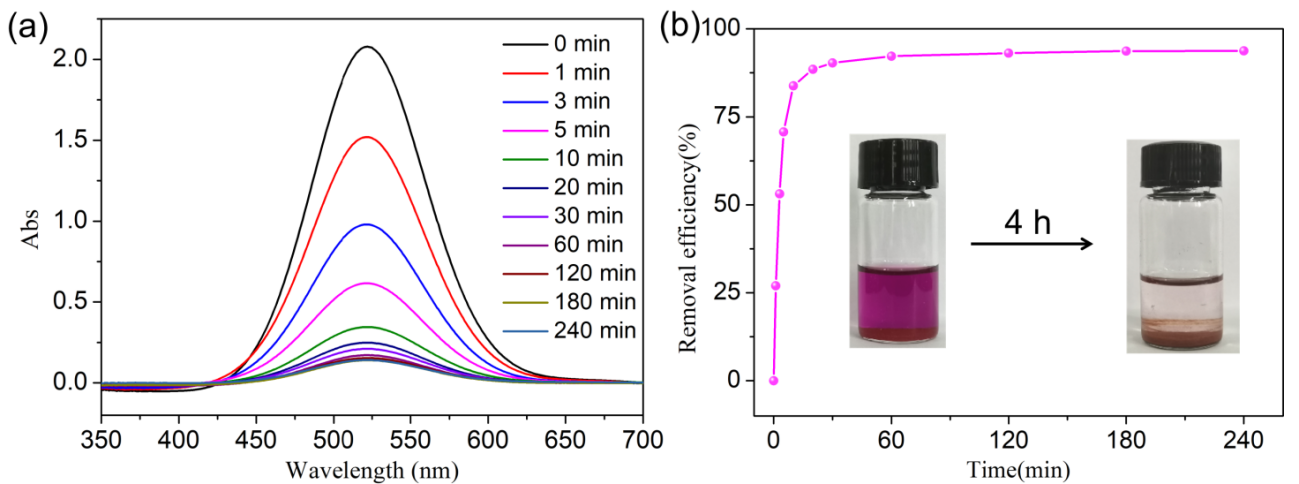


**Supplementary Figure S8.** (A) Temporal evolution of UV-vis absorption spectra for the adsorption of iodine by **NS-1** in cyclohexane and (B) the rate of iodine adsorption by **NS-1** in cyclohexane (*C*_0_ = 600 mg/L).


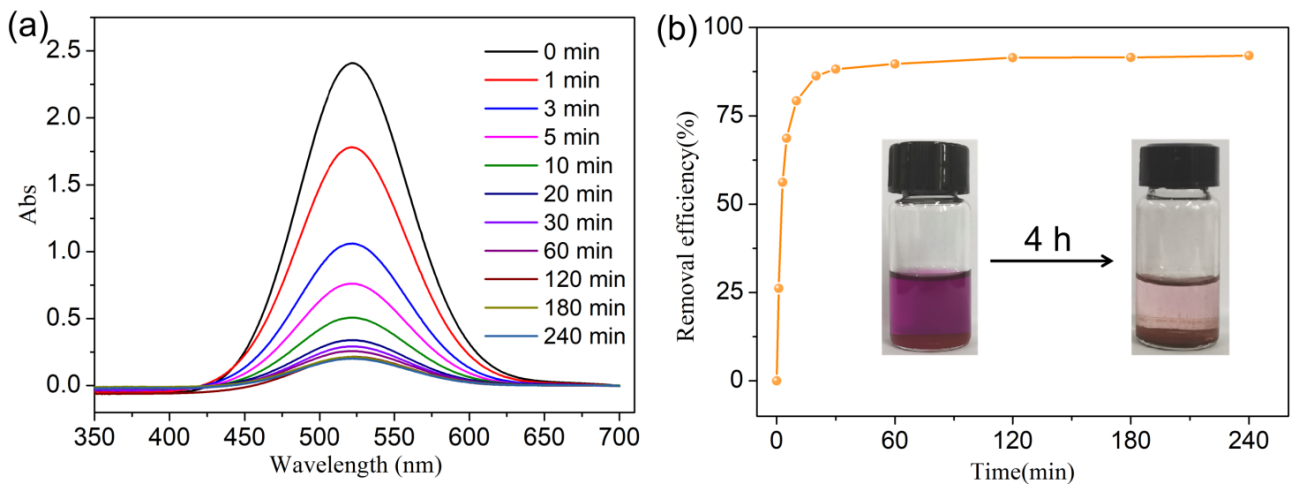


**Supplementary Figure S9.** (A) Temporal evolution of UV-vis absorption spectra for the adsorption of iodine by **NS-1** in cyclohexane and (B) the rate of iodine adsorption by **NS-1** in cyclohexane (*C*_0_ = 700 mg/L).


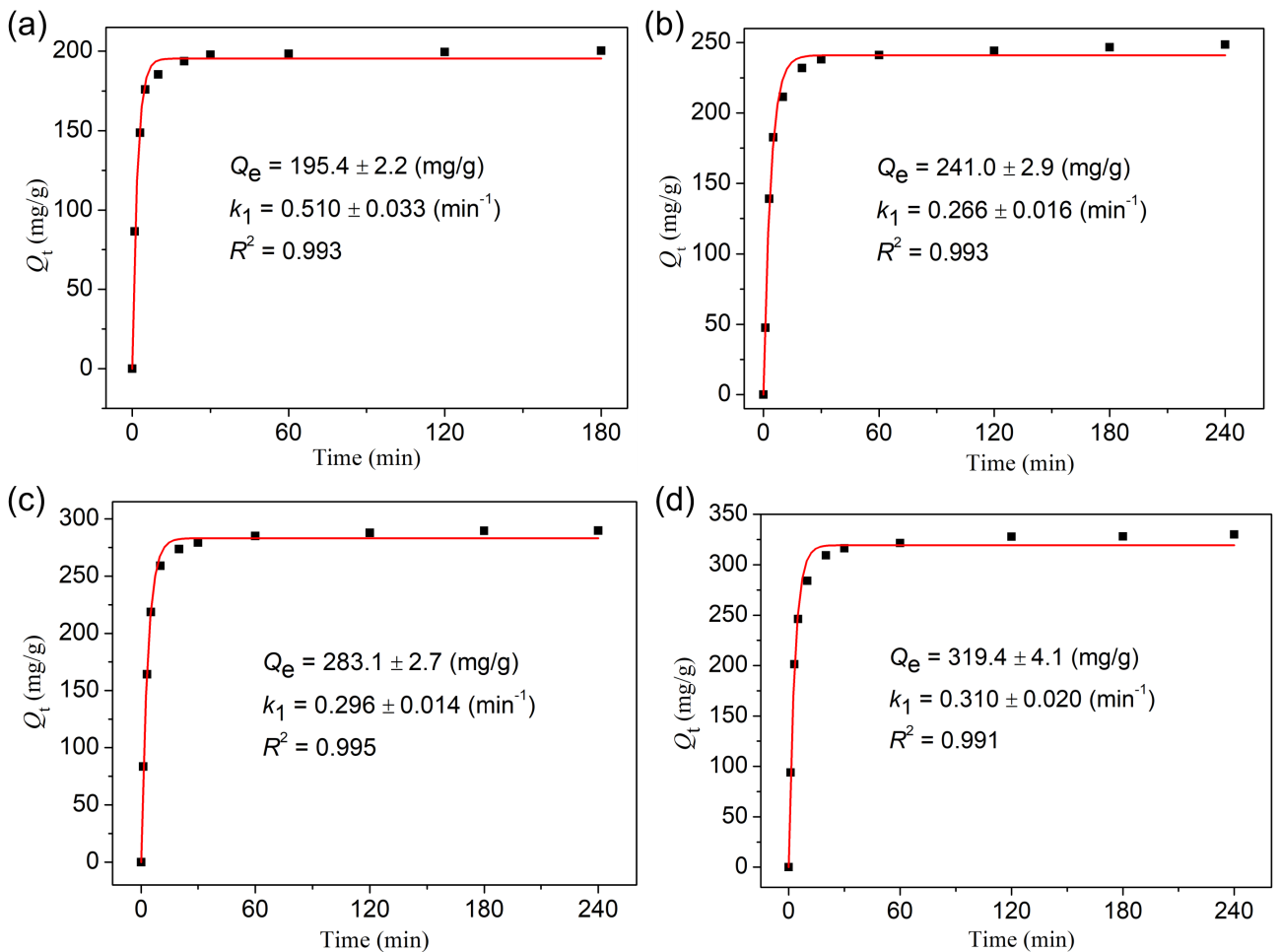


**Supplementary Figure S10.** The pseudo-first order kinetic models for the iodine adsorption kinetics of **NS-1** with the different initial concentrations of (A) 400 mg/L, (B) 500 mg/L, (C) 600 mg/L and (D) 700 mg/L cyclohexane solution.

**
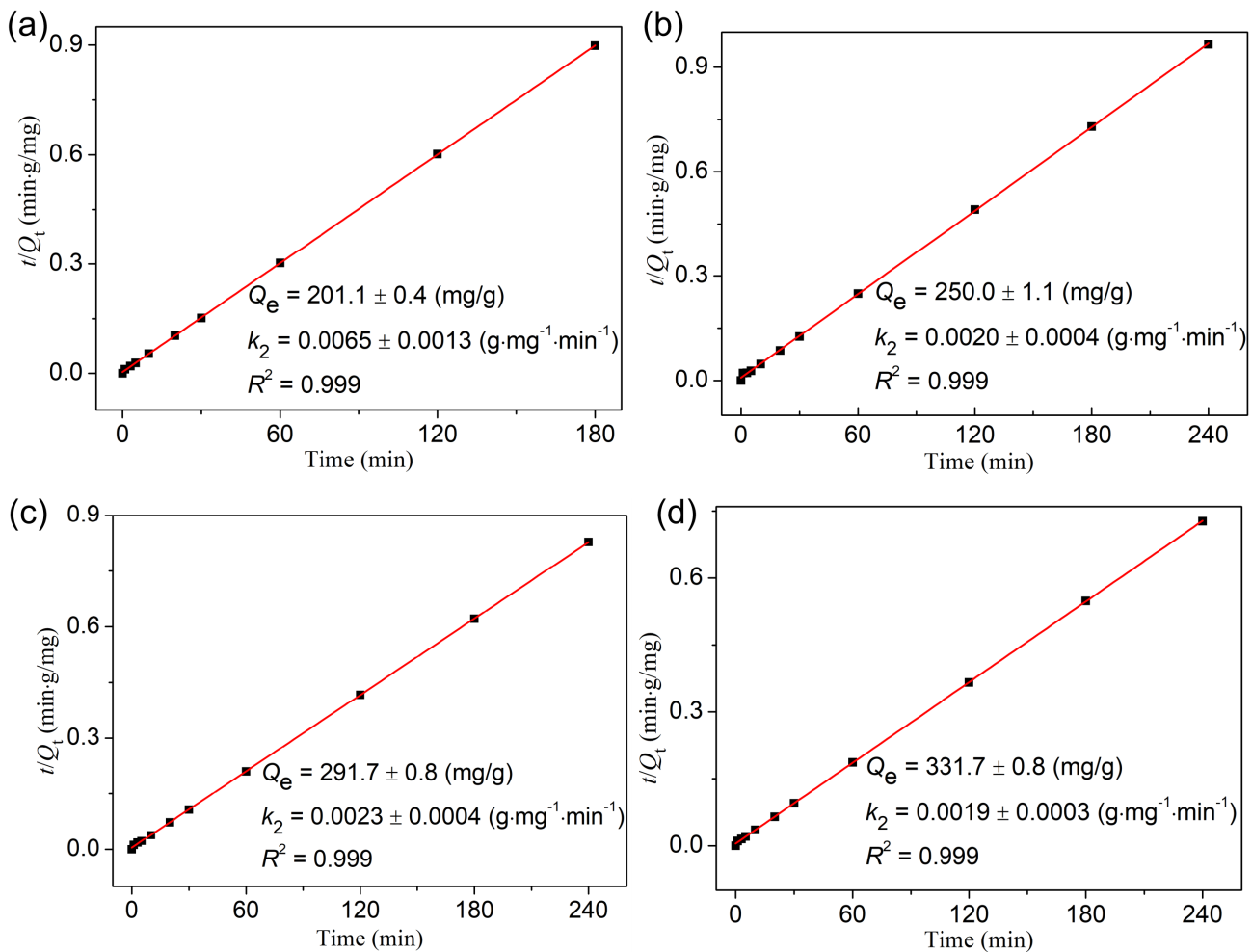
**

**Supplementary Figure S11.** The pseudo-second order kinetic models for the iodine adsorption kinetics of **NS-1** with the different initial concentrations of (A) 400 mg/L, (B) 500 mg/L, (C) 600 mg/L and (D) 700 mg/L cyclohexane solution.


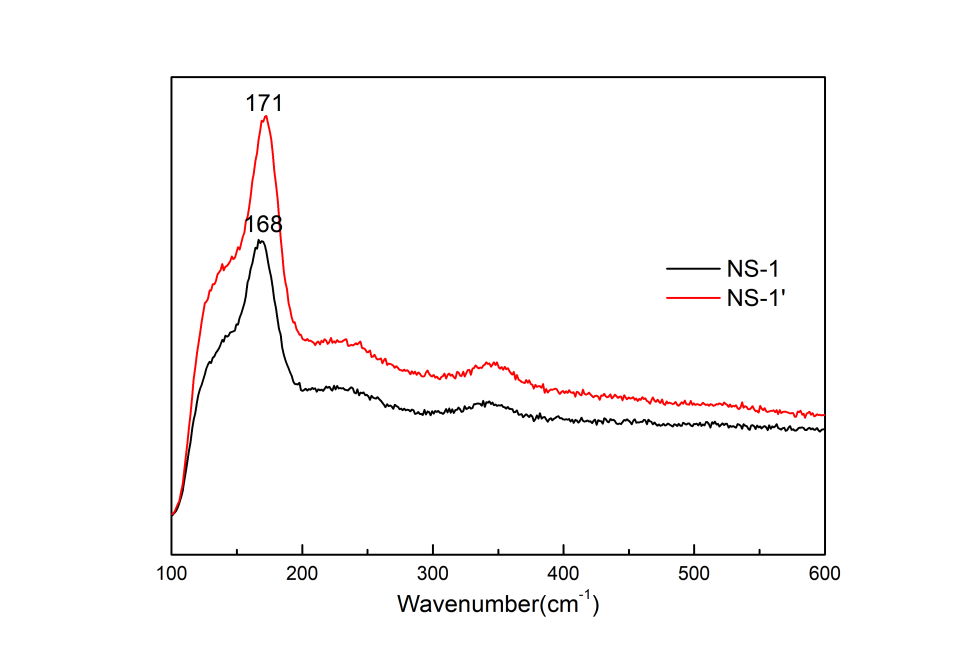


**Supplementary Figure S12.** Raman spectra of **NS-1** and **NS-1'**.


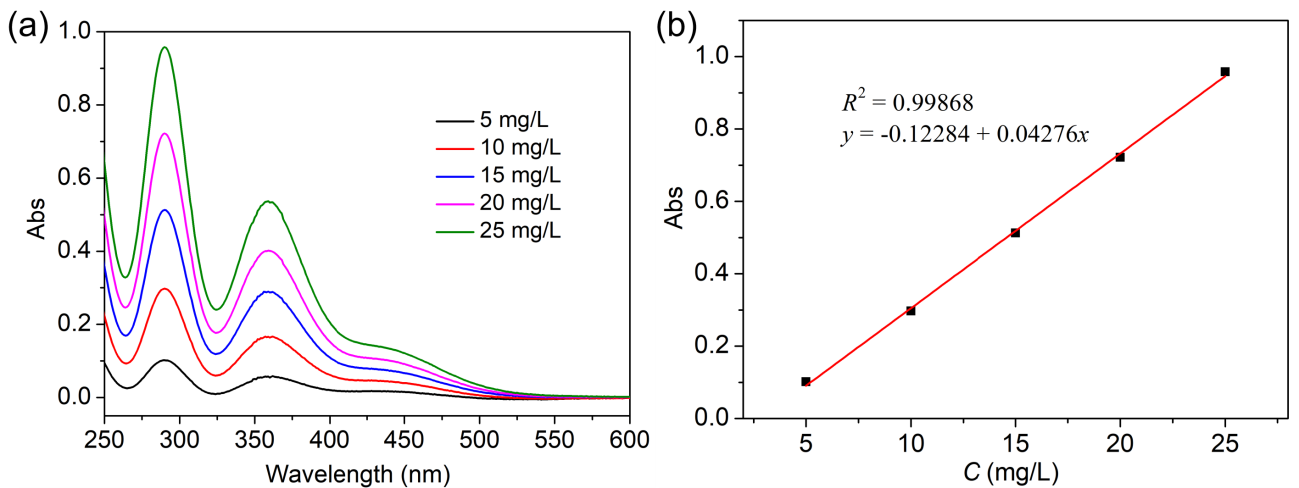


**Supplementary Figure S13.** The standard curve of iodine dissolved in EtOH.

**Supplementary Table S1.** Crystallographic data and structural refinement parameters for **NS-1**.

|  | **NS-1** |
| --- | --- |
| formula | Hf_3_Cu_4_I_4_C_24_H_16_N_4_O_16_ |
| formula weight | 1913.64 |
| temperature (*K*) | 293(2) |
| crystal system | tetragonal |
| space group | I4/mmm |
| *a* (Å) | 17.3742(11) |
| *b* (Å) | 17.3742(11) |
| *c* (Å) | 34.8923(15) |
| *α* (deg) | 90.00 |
| *β* (deg) | 90.00 |
| *γ* (deg) | 90.00 |
| Volume (Å^3^) | 10532.7(14) |
| Z | 4 |
| *D*_calc_ (g cm^-3^) | 1.207 |
| *μ* (mm^‑1^) | 4.934 |
| *F*(000) | 3440.0 |
| no. of rflns collected | 15958 |
| no. of indep rflns | 2646 [R_int_ = 0.0761, R_sigma_ = 0.0561] |
| GOF on F^2^ | 1.076 |
| *R*_1_, *wR*_2_ [*I* ≧2*σ* (*I*)] | *R*_1_ = 0.0838, *wR*_2_ = 0.2011 |
| *R*_1_, *wR*_2_ (all data) | *R*_1_ = 0.1437, *wR*_2_ = 0.2817 |
| $R_{1}=\sum\left\vert\left\vert F_{\text{o}}\right\vert-\left\vert F_{\text{c}}\right\vert\right\vert/\sum\left\vert F_{\text{o}}\right\vert$ and $wR_{2}=\left\{\sum\left[w(F_{\text{O}}^{2}-F_{\text{C}}^{2})^{2}\right]/\sum[w(F_{\text{O}}^{2})^{2}\right\}^{1/2}$ | |

**Supplementary Table S2.** The equilibrium adsorption amount and removal efficiency of iodine in cyclohexane solution with the different initial concentrations using **NS-1**.

| *C*_0_ (mg/L) | *Q_e_* (mg/g) | Removal efficiency (%)^a^ |
| --- | --- | --- |
| 300 | 147 | 99.1 |
| 400 | 200 | 96.8 |
| 500 | 248 | 95.4 |
| 600 | 289 | 93.7 |
| 700 | 329 | 92.0 |

a: The equilibrium removal efficiency of iodine in **NS-1**.

**Supplementary Table S3.** Summary of the parameters from Langmuir and Freundlich isotherm models for the iodine adsorption in cyclohexane via **NS-1**.

| Langmuir isotherm model | | | Freundlich isotherm model | | |
| --- | --- | --- | --- | --- | --- |
| *Q*_max_ (mg/g) | *K*_L_(L/mg) | *R*^2^ | *K*_F_ (mg/g) | 1/*n* | *R*^2^ |
| 320.5 | 0.220 | 0.748 | 104.1 | 0.28 | 0.976 |
