# Supplementary material for: Heterometallic Metal-Organic Framework Based on [Cu4I4] and [Hf6O8] Clusters for Adsorption of Iodine
Source: Front Chem. 2022 Apr 29;10:864131. doi: 10.3389/fchem.2022.864131 (PMC9098963; doi:10.3389/fchem.2022.864131)
Supplement: Supplementary file 1 [file DataSheet1.zip › Data Sheet 1/Supplementary_Material.pdf]

Supplementary Material

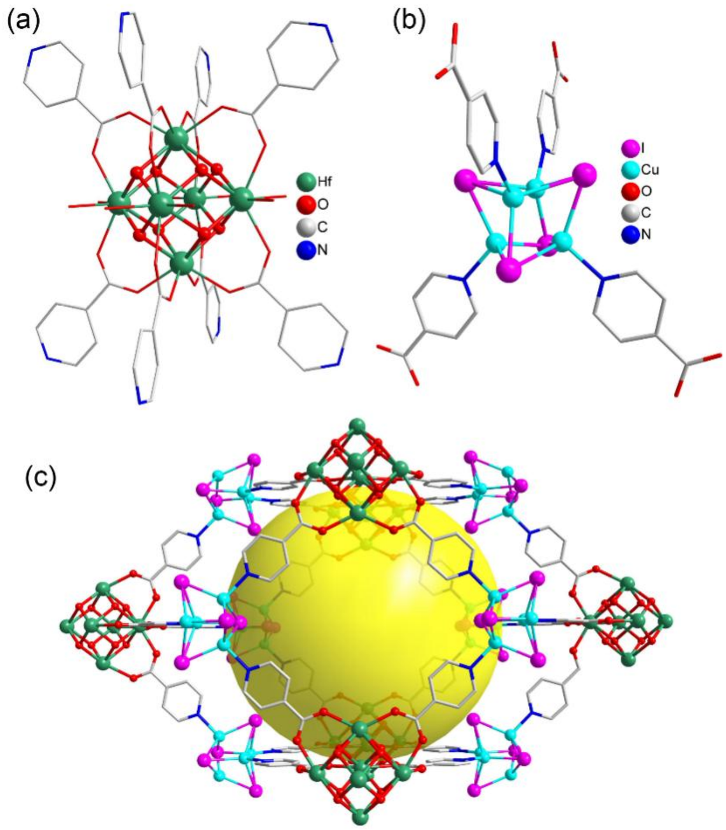

Supplementary Figure S1. (A) The 8-connected [Hf_6(μ_3-OH)$_8$(OH)$_8$(ina)$_8$] cluster, (B) the 4-connected [Cu_4I_4(ina)$_4$]$^{4-}$ cluster and (C) the cage built by six [Hf_6(μ_3-OH)$_8$(OH)$_8$]$^{8+}$ clusters, eight [Cu_4I_4] clusters and twenty-four ina$^-$ linkers in **NS-1**.

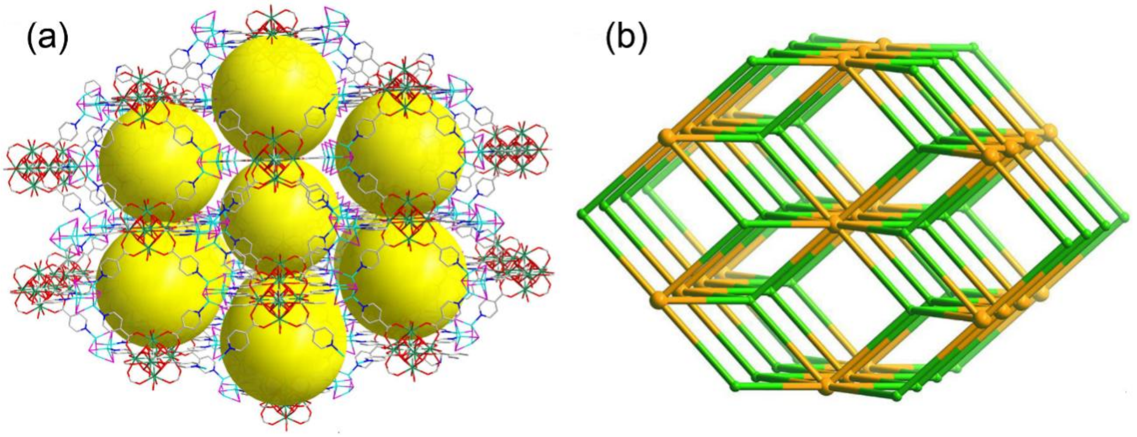

Supplementary Figure S2. (A) The 3D network and (B) The **flu** topology of **NS-1**.

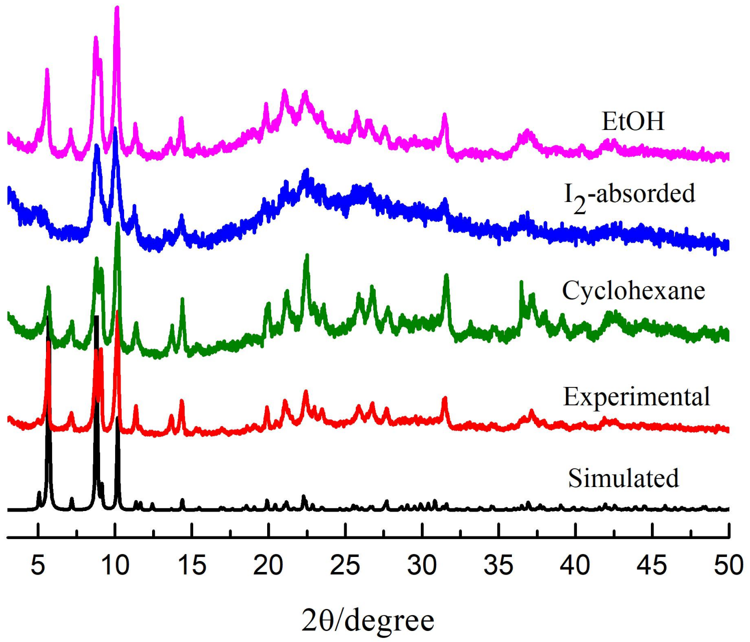

Supplementary Figure S3. Powder X-ray diffraction patterns of simulated and as-synthesized **NS-1**, **NS-1** after treated in cyclohexane, iodine-dissolved cyclohexane solution with 300 mg/L and **NS-1'** after treated in EtOH solution.

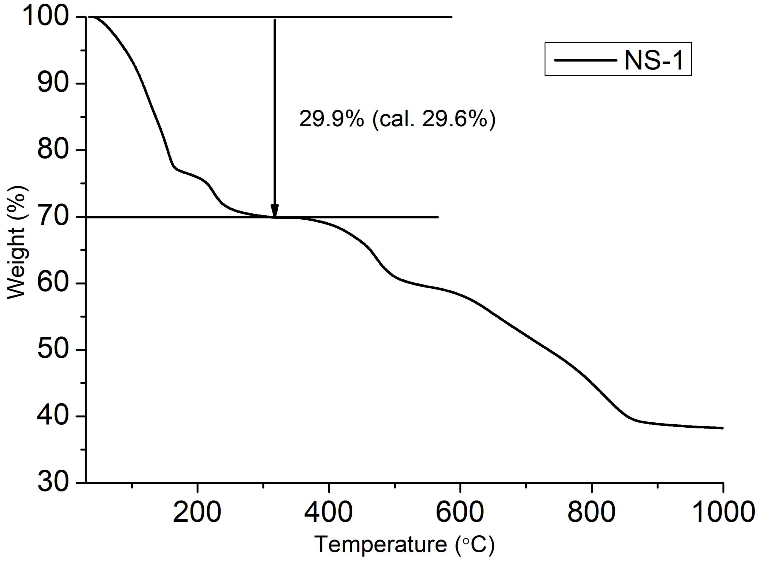

Supplementary Figure S4. Thermogravimetric analysis of **NS-1**.

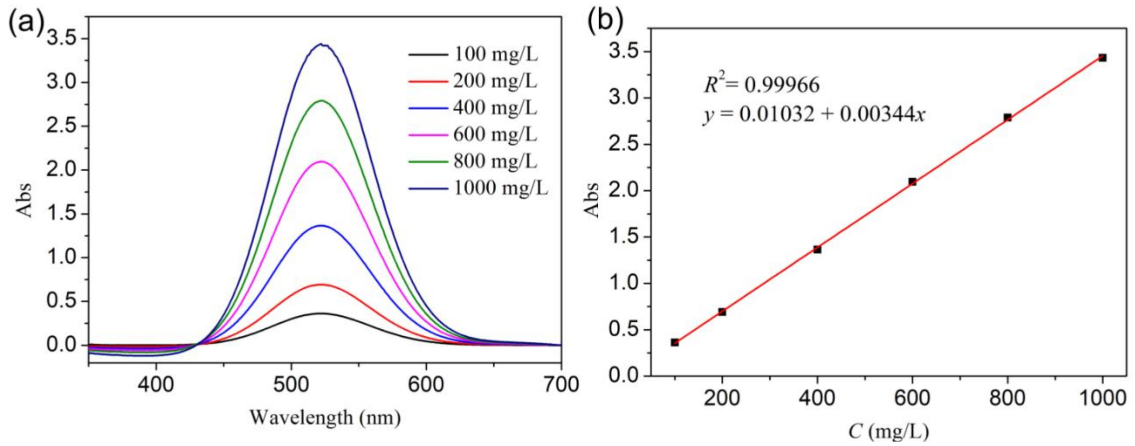

Supplementary Figure S5. The standard curves of iodine-dissolved in cyclohexane solution.

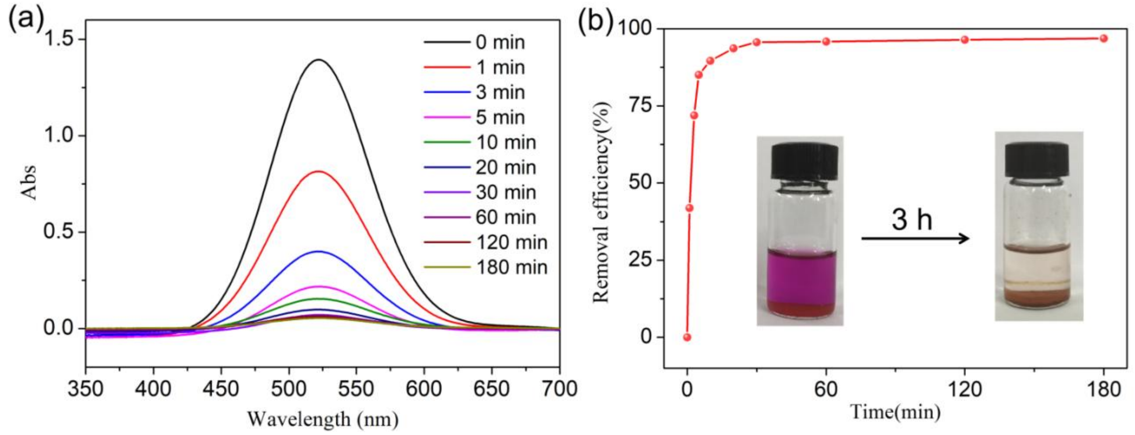

Supplementary Figure S6. (A) Temporal evolution of UV-vis absorption spectra for the adsorption of iodine by **NS-1** in cyclohexane and (B) the rate of iodine adsorption by **NS-1** in cyclohexane (C_0 = 400 mg/L).

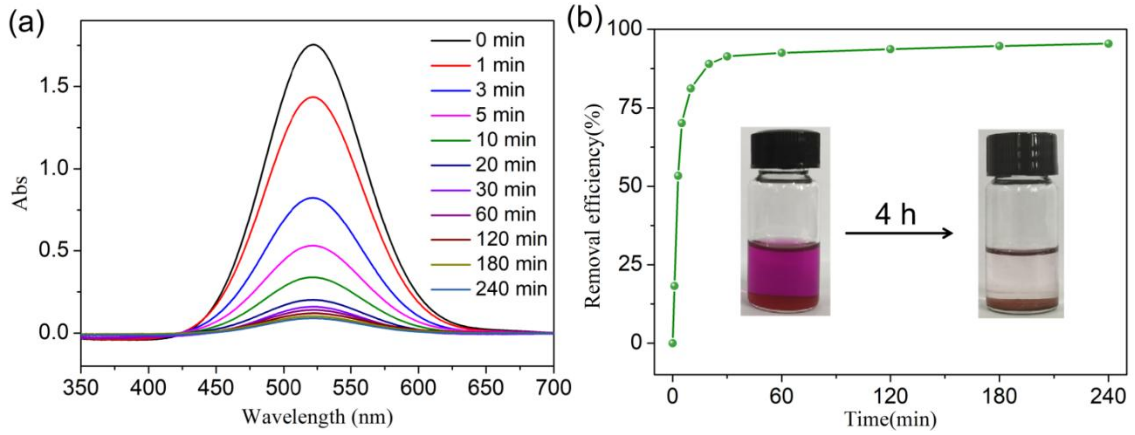

Supplementary Figure S7. (A) Temporal evolution of UV-vis absorption spectra for the adsorption of iodine by **NS-1** in cyclohexane and (B) the rate of iodine adsorption by **NS-1** in cyclohexane (C_0 = 500 mg/L).

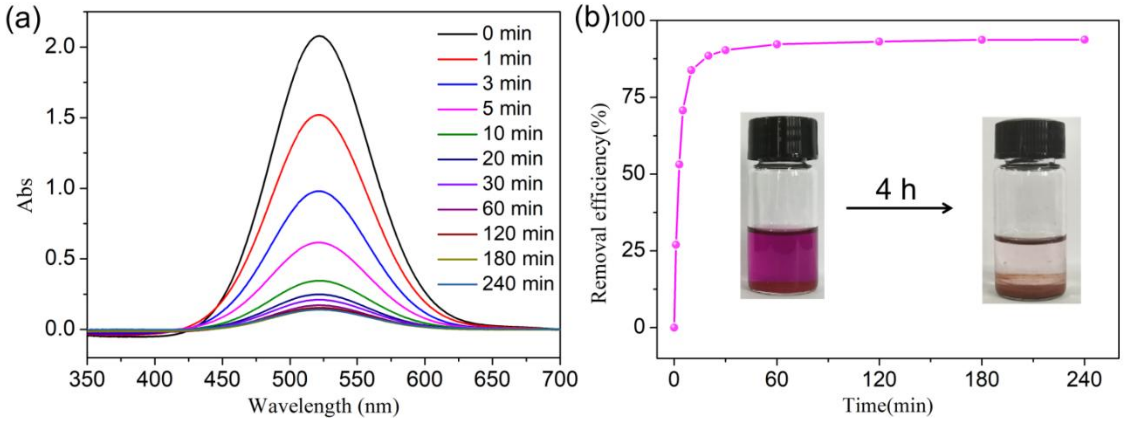

Supplementary Figure S8. (A) Temporal evolution of UV-vis absorption spectra for the adsorption of iodine by **NS-1** in cyclohexane and (B) the rate of iodine adsorption by **NS-1** in cyclohexane (C_0 = 600 mg/L).

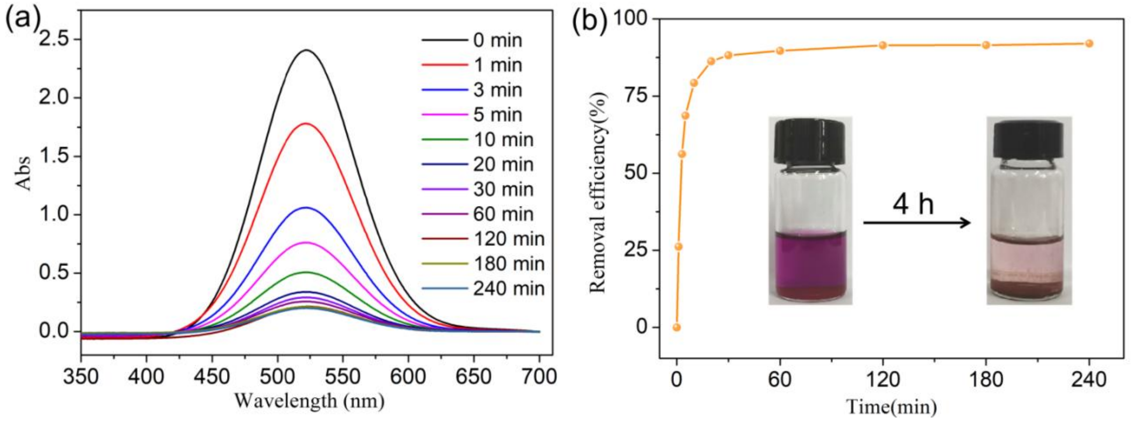

Supplementary Figure S9. (A) Temporal evolution of UV-vis absorption spectra for the adsorption of iodine by **NS-1** in cyclohexane and (B) the rate of iodine adsorption by **NS-1** in cyclohexane (C_0 = 700 mg/L).

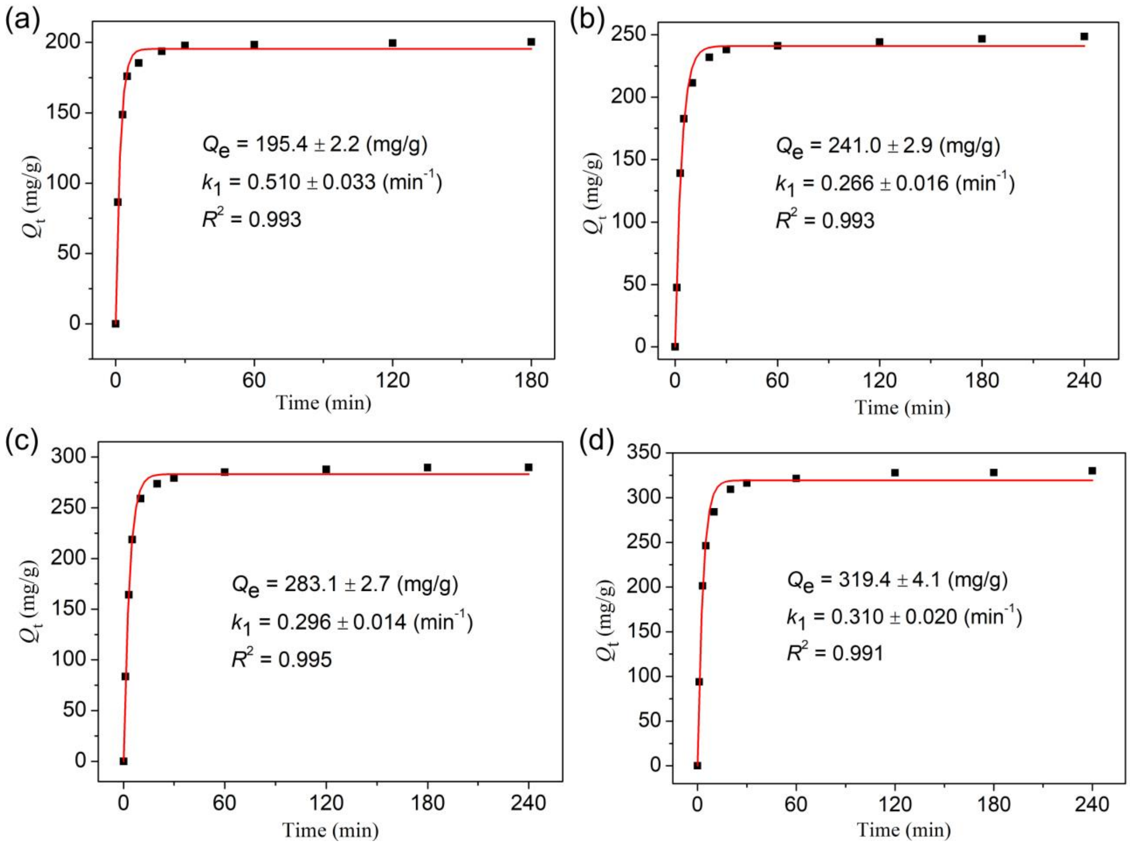

Supplementary Figure S10. The pseudo-first order kinetic models for the iodine adsorption kinetics of **NS-1** with the different initial concentrations of (A) 400 mg/L, (B) 500 mg/L, (C) 600 mg/L and (D) 700 mg/L cyclohexane solution.

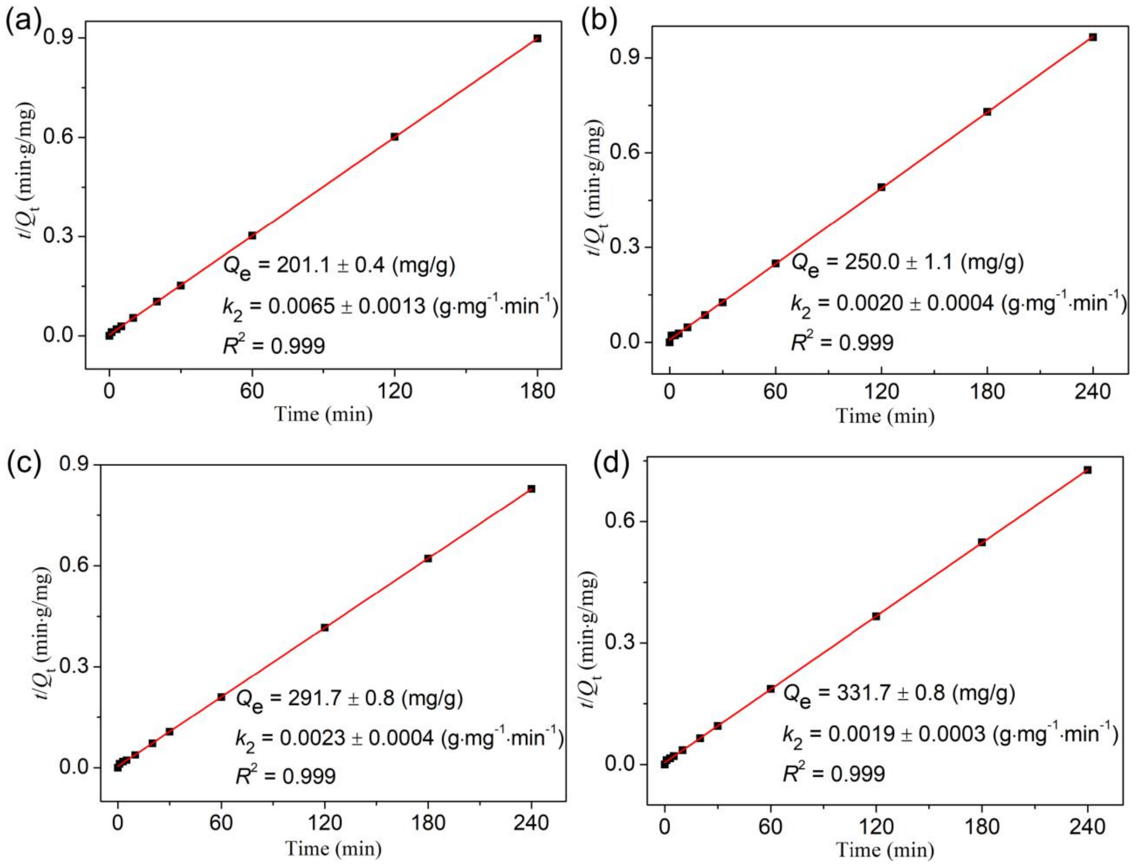

Supplementary Figure S11. The pseudo-second order kinetic models for the iodine adsorption kinetics of **NS-1** with the different initial concentrations of (A) 400 mg/L, (B) 500 mg/L, (C) 600 mg/L and (D) 700 mg/L cyclohexane solution.

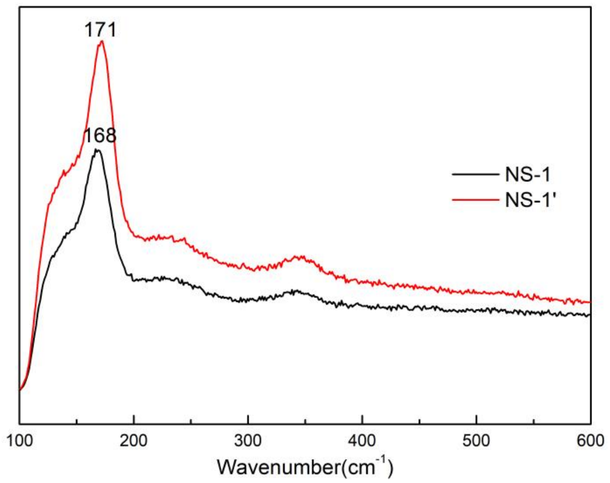

Supplementary Figure S12. Raman spectra of **NS-1** and **NS-1'**.

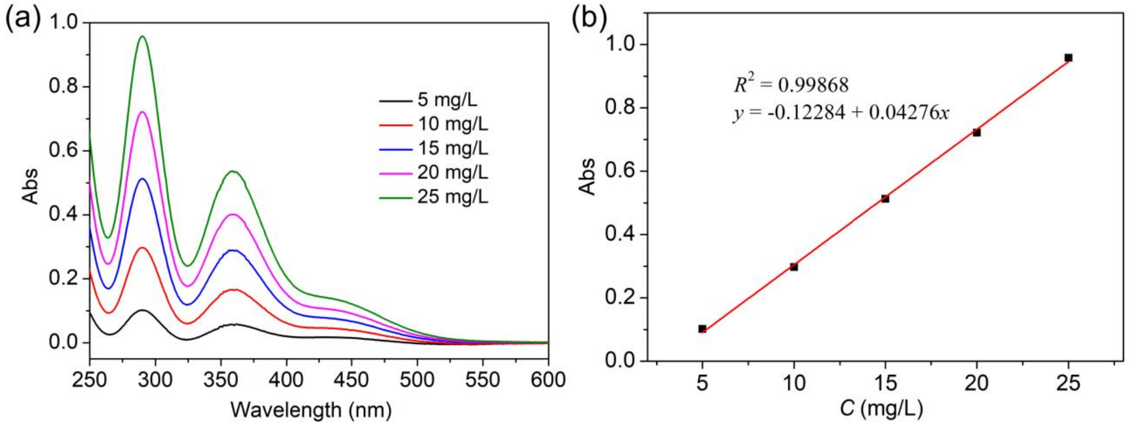

Supplementary Figure S13. The standard curve of iodine dissolved in EtOH.

Supplementary Table S1. Crystallographic data and structural refinement parameters for **NS-1**.

	**NS-1**
formula	$Hf_3Cu_4I_4C_{24}H_{16}N_4O_{16}$
formula weight	1913.64
temperature (*K*)	293(2)
crystal system	tetragonal
space group	I4/mmm
a (Å)	17.3742(11)
b (Å)	17.3742(11)
c (Å)	34.8923(15)
α (deg)	90.00
β (deg)	90.00
γ (deg)	90.00
Volume (Å^3)	10532.7(14)
Z	4
D_{calc} (g cm^{-3})	1.207
μ (mm^{-1})	4.934
F(000)	3440.0
no. of rflns collected	15958
no. of indep rflns	2646 [R_{int} = 0.0761, R_{sigma} = 0.0561]
GOF on F^2	1.076
R_1, wR_2 [$I \geqq 2\sigma(I)$]	R_1 = 0.0838, wR_2 = 0.2011
R_1, wR_2 (all data)	R_1 = 0.1437, wR_2 = 0.2817
$R_1 = \sum \|\|F_o\| - \|F_c\|\| / \sum \|F_o\|$ and $wR_2 = \{\sum [w(F_O^2 - F_C^2)^2] / \sum [w(F_O^2)^2\}^{1/2}$	

Supplementary Table S2. The equilibrium adsorption amount and removal efficiency of iodine in cyclohexane solution with the different initial concentrations using **NS-1**.

C_0 (mg/L)	Q_e (mg/g)	Removal efficiency (%)[a]
300	147	99.1
400	200	96.8
500	248	95.4
600	289	93.7
700	329	92.0

a: The equilibrium removal efficiency of iodine in **NS-1**.

Supplementary Table S3. Summary of the parameters from Langmuir and Freundlich isotherm models for the iodine adsorption in cyclohexane via **NS-1**.

Langmuir isotherm model			Freundlich isotherm		
Q_{max} (mg/g)	K_L(L/mg)	R^2	K_F (mg/g)	$1/n$	R^2
320.5	0.220	0.748	104.1	0.28	0.976
